# Genomic insights into the endangered white-eared night heron (*Gorsachius magnificus*)

**DOI:** 10.1186/s12863-024-01194-1

**Published:** 2024-01-30

**Authors:** Haoran Luo, Qingxian Lin, Wenzhen Fang, Xiaolin Chen, Xiaoping Zhou

**Affiliations:** Key Laboratory of the Ministry of Education for Coastal and Wetland Ecosystems, College of the Environment and Ecology, Xiamen University, 361102 Xiamen, China

**Keywords:** The white-eared night heron, Endangered bird, Genome assembly, Genome annotation, Heterozygosity

## Abstract

**Objectives:**

A genome sequence of a threatened species can provide valuable genetic information that is important for improving the conservation strategies. The white-eared night heron (*Gorsachius magnificus*) is an endangered and poorly known ardeid bird. In order to support future studies on conservation genetics and evolutionary adaptation of this species, we have reported a *de novo* assembled and annotated whole-genome sequence of the *G. magnificus*.

**Data description:**

The final draft genome assembly of the *G. magnificus* was 1.19 Gb in size, with a contig N50 of 187.69 kb and a scaffold N50 of 7,338.28 kb. According to BUSCO analysis, the genome assembly contained 97.49% of the 8,338 genes in the Aves (odb10) dataset. Approximately 10.52% of the genome assembly was composed of repetitive sequences. A total of 14,613 protein-coding genes were predicted in the genome assembly, with functional annotations available for 14,611 genes. The genome assembly exhibited a heterozygosity rate of 0.49 heterozygosity per kilobase pair. This draft genome of *G. magnificus* provides valuable genomic resources for future studies on conservation and evolution.

## Objectives

The white-eared night heron (*Gorsachius magnificus*) is a medium-sized ardeid bird that distributes in tropical and subtropical moist lowland forests in southern and southwestern China [1–3], northern Vietnam [4], and northeastern India [5]. Due to its small and fragmented population, the *G. magnificus* is currently listed as an Endangered (EN) species by the International Union for the Conservation of Nature (IUCN) [6] and is listed in the first order of the National Key Protected Wild Animal List in China [7].

The *G. magnificus* is not well understood due to its solitary, nocturnal, and cryptic behaviour [4, 8]. Previous studies on the *G. magnificus* primarily focused on documenting its distribution in new areas [2, 4, 9]. These findings expanded our knowledge of the *G. magnificus*’ range and resulted in its threat category being changed from Critically Endangered to Endangered by IUCN in 2000 [10]. Some researchers have suggested further downgrading its threat status because the *G. magnificus* has been observed in almost the entire southern region of China [11]. However, ecological niche modelling predicted that suitable habitats for the *G. magnificus* are limited and scattered within the mountain chains of southern China [12]. It also suggested that future climate change could alter its distribution and pose a threat to its population. Therefore, the authors recommended exercising caution when considering any downgrading of the threat status for the *G. magnificus*.

The genetic information of a threatened species is valuable for various aspects of their conservation biology, including estimating effective population size, inbreeding level, genetic diversity, and population structure [13]. However, up until now, very little genetic information has been available for *G. magnificus*, except for the sequence of complete mitochondrial DNA [14]. In this study, we sequenced the genome of the *G. magnificus* and evaluated genetic diversity using heterozygous single nucleotide polymorphisms (SNPs) at the whole genome level. The genetic diversity of the *G. magnificus* can help to correctly assess its conservation status. Additionally, the draft genome sequence can facilitate future conservation biology studies to help protect this endangered species.

## Data description

A muscle sample of a dead *G*. *magnificus* was provided by the Jiulingshan National Reserve, which was found and confiscated by the reserve personnel from a local farmers’ market in Jing’an Town (115.11’ 25” E, 28.69’ 21’’N), Yichun City, Jiangxi province, China in 2007. The muscle tissue was stored at -80 ℃ after collection. This research was approved by the Ethics Committee for Animal Experimentation of the Xiamen University. Genomic DNA was extracted using the QIAGEN® Puregene Tissue Core Kit A (Qiagen, Beijing, China), following the manufacturer’s instructions. Two short insert libraries (230 and 500 bp) were prepared using the Illumina TruSeq DNA Library Preparation Kit (Illumina, San Diego, USA), while three mate pair libraries (2, 5, and 10 kb) were constructed using the Nextera Mate Pair Sample Preparation Kit (Illumina, San Diego, USA). The libraries were sequenced on the Illumina HiSeq 2500 sequencing platform at Novogene (Beijing). The sequencing depths were 30.30× and 26.10× for the two short insert libraries, and 16.05×, 11.26× and 9.90× for the three mate pair libraries, respectively. Raw reads were filtered using Cutadapt [15] and Trimmomatic [16] to remove adapters and low-quality reads (quality score < 20), respectively. The resulting clean reads were assembled using SOAPdenovo2 following the official guidelines [17]. The estimated genome size was determined using 17 k-mers with Jellyfish [18]. A total of 123.14 Gb of clean sequence data was obtained, and the estimated genome size was approximately 1.32 Gb based on the 17 K-mer distribution. The final genome assembly had a length of 1.19 Gb, with contig and scaffold N50 sizes of 187.69 kb and 7,338.28 kb, respectively (Table 1, Data file 1). The longest scaffold was 29,902.37 kb. The completeness of the genome assembly was assessed using the Benchmarking Universal Single-Copy Orthologs (BUSCO) v5.1.3 [19] with the Aves (odb10) dataset. The analysis showed that 97.49% of the BUSCO genes (8103 single-copy and 25 duplicated) were complete, 0.56% were fragmented (47 genes), and 1.95% were missing (163 genes) (Table 1, Data file 2).

**Table 1:** Overview of data files/data sets

Label	Name of data file/data set	File types and extension	Data repository and identifier (DOI or accession number)
Data file 1 Data file 2 Data set 1 Data set 2 Data file 3 Data file 4 Data file 5	Statistics of sequencing depths, contigs, scaffolds and GC content of the *G*. *magnificus* genome assembly Statistics of complete, fragmented and missing BUSCOs of the *G*. *magnificus* genome assembly Sequencing reads of *G*. *magnificus* genomic DNA Genome assembly of *G*. *magnificus* Comparison of the heterozygosity of *G*. *magnificus* and its close related species Structure annotation gene model file Gene model functional annotation files	Spreadsheet (.xlsx) Text raw output (.txt) Fastq files (.fq.gz) Fasta file (.fa) Portable document format (.pdf) Gff file (.gff) Zipped (compressed) file (.zip)	Figshare: 10.6084/m9.figshare.24311935[28] Figshare: 10.6084/m9.figshare.24311935 [28] NCBI SRA Database: SRP364708 https://identifiers.org/insdc.sra:SRP364708 [29] NCBI GenBank Database: JALHKT000000000 https://identifiers.org/nucleotide:JALHKT000000000 [30] Figshare: 10.6084/m9.figshare.24311935, [28] Figshare: 10.6084/m9.figshare.24311935, [28] Figshare: 10.6084/m9.figshare.24311935, [28]

RepeatModeler and RepeatMasker (http://www.repeatmasker.org) with the Repbase2 repeat database were used to identify interspersed repeats using a de novo and homology-based approach with default parameters, respectively. TRF [20] was used to identify tandem repeats. The results showed that approximately 10.52% of the assembled genome consisted of repetitive sequences. This included 0.72% tandem repeat sequences, 0.62% DNA repeat elements, 6.07% long interspersed nuclear elements (LINE), 0.11% short interspersed nuclear elements (SINE), 1.67% long terminal repeat elements (LTR), and 1.33% unknown repetitive sequences. A total of 14,613 protein-coding genes were predicted using Genewise2 [21], with an average coding sequence (CDS) length of 1417.22 bp. Out of these, 14,611 (99.99%) protein-coding genes were functionally annotated by searching against EggNog-mapper V2.1.2 [22], KEGG [23], GO [24], SwissProt [25], and TrEMBL [26] databases. Additionally, the assembled genome also contained 368 candidate microRNA genes (31,150 bp, 0.0026% of the genome) and 230 candidate tRNAs (17,191 bp, 0.0014% of the genome). The genetic diversity of the *G. magnificus* was assessed using individual heterozygosity from SNPs’ genotypes, which indicated a genetic diversity of 0.49 heterozygosity per kilobase pair (578,569 heterozygosity loci) (Table 1, Data file 3). This heterozygosity was extremely low compared to other ardeid birds. For example, the heterozygosity of the Japanese night heron (*G. goisagi*, VU), the little egret (*Egretta garzetta*), the boat-billed heron (*Cochlearius cochlearius*, LC) and the black crown night heron (*Nycticorax nycticorax*) were 0.83, 2.51, 4.88 and 6.25 per kilobase pair, respectively. Additionally, the heterozygosity of the *G. magnificus* was comparable to the Crested ibis *Nippon nippon*, a species that faced near-extinction and rebirth, with a genetic diversity of 0.43 heterozygosity per kilobase pair [27]. The low genome-wide heterozygosity observed in other endangered bird species has been shown to be associated with inbreeding depression and the accumulation of harmful mutations [27]. Therefore, we suggested that the endangered conservation status of *G. magnificus* be maintained, and that greater emphasis be placed on restoring its genetic diversity in future conservation efforts.

In conclusion, the draft genome sequence of *G. magnificus* can serve as a valuable resource for future studies on the genetic mechanisms underlying adaptations and the estimation of important parameters relevant to conservation efforts.

## Limitations

Due to the difficulty of obtaining fresh tissues, this draft genome assembly was generated using short-read shotgun sequencing. Consequently, the final genome assembly size was smaller than the K-mer estimation, suggesting that the assembly contained a degree of fragments and gaps. In the future, if fresh tissues could be collected, long-read sequencing technologies, such as Pacific BioSciences (PacBio) or Oxford Nanopore sequencing, would help to improve the completeness and accuracy of the genome assembly.

## Data Availability

The draft genome assembly data are available at GenBank with the accession number: JALHKT000000000 (https://identifiers.org/nucleotide:JALHKT000000000) [29]. The associated BioProject number is PRJNA816834 (https://www.ncbi.nlm.nih.gov/bioproject/PRJNA816834/) [30]. The genomic annotation flies for genomic analyses can be found at Figshare repository (https:///10.6084/m9.figshare.24311935) [28].

## Abbreviations

SNPs: Single nucleotide polymorphisms
BUSCO: Benchmarking Universal Single-Copy Orthologs
IUCN: International Union for the Conservation of Nature
CDS: Coding sequence
LINE: Long interspersed nuclear elements
SINE: Short interspersed nuclear elements
LTR: Long terminal repeat elements
PacBio: Pacific BioSciences
